# Pediatric hypertrophic cardiomyopathy caused by a novel *TNNI3* variant

**DOI:** 10.1038/s41439-024-00272-1

**Published:** 2024-03-29

**Authors:** Natsuko Inagaki, Tomoya Okano, Masatake Kobayashi, Masatsune Fujii, Yoshinao Yazaki, Yasuyoshi Takei, Hisanori Kosuge, Shinji Suzuki, Takeharu Hayashi, Masahiko Kuroda, Kazuhiro Satomi

**Affiliations:** 1 Department of Cardiology, Tokyo Medical University, Tokyo, Japan; 2 Department of Clinical Genetics Center, Tokyo Medical University, Tokyo, Japan; 3 Department of Pediatrics and Adolescent Medicine, Tokyo Medical University, Tokyo, Japan; 4 Department of Physiology, Tokai University School of Medicine, Isehara, Japan; 5 Department of Molecular Pathology, Tokyo Medical University, Tokyo, Japan

**Keywords:** Cardiomyopathies, Medical genetics

## Abstract

*TNNI3* is a gene that causes hypertrophic cardiomyopathy (HCM). A 14-year-old girl who was diagnosed with nonobstructive HCM presented with cardiopulmonary arrest due to ventricular fibrillation. Genetic testing revealed a novel de novo heterozygous missense variant in *TNNI3*, NM_000363.5:c.583A>T (p.Ile195Phe), which was determined to be the pathogenic variant. The patient exhibited progressive myocardial fibrosis, left ventricular remodeling, and life-threatening arrhythmias. Genetic testing within families is useful for risk stratification in pediatric HCM patients.

Hypertrophic cardiomyopathy (HCM) is characterized by left ventricular (LV) hypertrophy and diastolic dysfunction, and it is the most common inherited cardiovascular disease. Approximately half of patients with genetically identified HCM have a family history of the disease, which follows an autosomal dominant mode of inheritance. Many disease-causing pathological variants have been identified in genes encoding sarcomere proteins, which are the contractile units of the myocardium. The prevalence of HCM is estimated to be at least 1 in 500 in the general population^1,2^, and advancements in diagnostic methods such as imaging and genetic analysis have increased its diagnosis. Recent research suggests that 1 in 200 people may have HCM^3^.

Nonobstructive HCM is common among patients with HCM and is typically well tolerated. However, a small number of patients may experience disease progression, which is characterized by LV remodeling, leading to thinning of the LV wall with diffuse replacement scarring; in some cases, sudden cardiac death may occur as the initial manifestation^4–6^. A previous study showed that patients diagnosed with HCM at a young age have a worse prognosis than those diagnosed as adults, with sudden death occurring approximately twice as often^2^. Therefore, early diagnosis and prediction of the severity of pediatric cardiomyopathy are crucial^2,7^. In this report, we present the case of a patient with a novel *TNNI3* heterozygous variant who was diagnosed with nonobstructive HCM and experienced progressive myocardial fibrosis, LV structural remodeling, and life-threatening arrhythmias during the follow-up period.

The patient was a 14-year-old girl who was asymptomatic and lacked any family history of cardiomyopathy, including sudden death or cardiac hypertrophy (Fig. 1A). Abnormal electrocardiographic findings were incidentally discovered during a routine school physical examination, and subsequent evaluation with echocardiogram and cardiac magnetic resonance imaging (MRI) confirmed the diagnosis of HCM. Echocardiography at the time of diagnosis revealed preserved LV contractility, with an LV ejection fraction of 65% and no evidence of abnormal wall motion, and heterogeneous wall thickening was observed primarily in the inferoseptal area, with a maximum wall thickness of 15 mm. Cardiac MRI examination did not show any evidence of myocardial edema or late gadolinium enhancement (LGE) (Fig. 1B, a–c). A treadmill exercise test conducted for risk assessment did not reveal any ventricular arrhythmias, and dobutamine stress echocardiography did not show any findings suggestive of an obstructed outflow tract. Consequently, she underwent regular follow-up without aggressive treatment, including drug therapy. Approximately one year into the follow-up period, she suffered cardiac arrest due to ventricular fibrillation while running up the stairs at school and was successfully resuscitated. Following transportation to the hospital, cardiac MRI revealed no change in the degree of wall thickening; however, new myocardial edema was detected in the thickened inferoseptal area and the anterolateral wall (Fig. 1B, d, e). Furthermore, LGE corresponded to the area of myocardial edema (Fig. 1B, f). During her hospital stay, she underwent subcutaneous implantable cardioverter-defibrillator (S-ICD) implantation, and beta-blockers were initiated. After 12 months, a stress test (involving cardiopulmonary and treadmill stress tests), echocardiogram, and cardiac MRI were performed, revealing no significant progression of her cardiac condition.

**Fig. 1:**
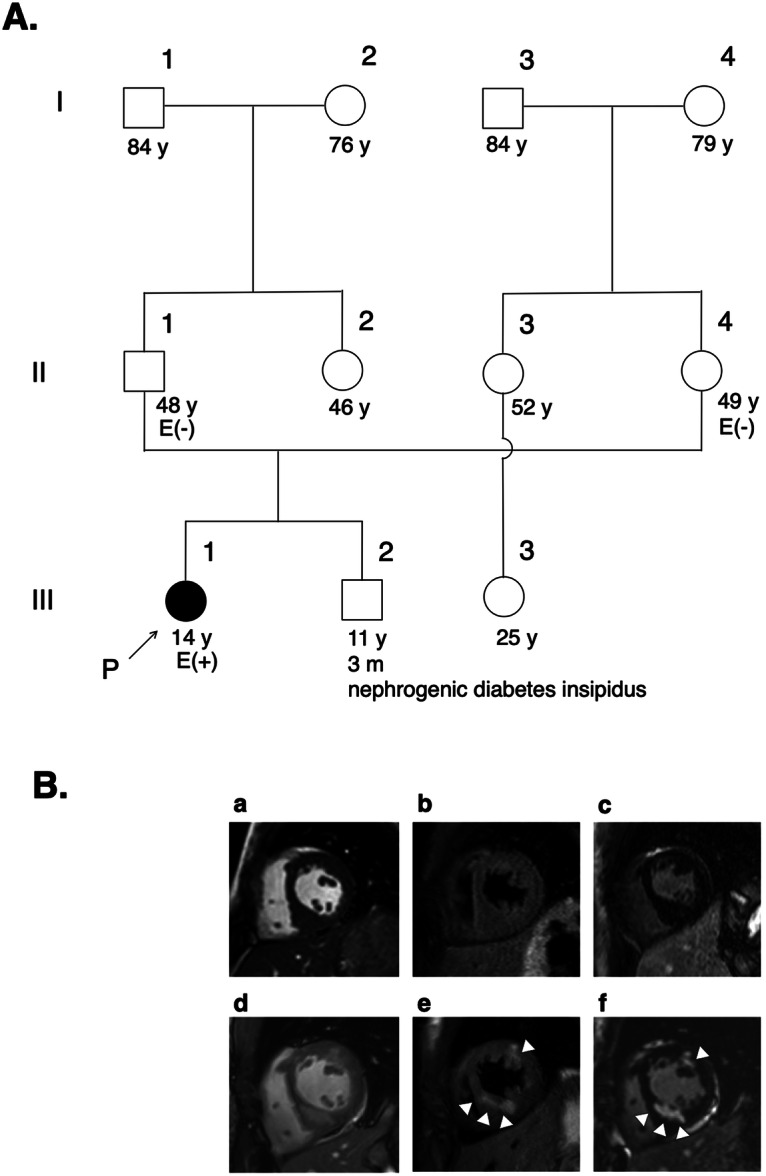
Family pedigree and Cardiac magnetic resonance. **A** Family pedigree showing the inheritance pattern of cardiomyopathy. Squares represent males, and circles represent females. The black symbols indicate affected individuals, and the open symbols indicate unaffected individuals. The arrow indicates the proband who was diagnosed with hypertrophic cardiomyopathy (HCM). P, proband; E, genetic evaluation; +, presence of *TNNI3* variant; −, absence of *TNNI3* variant. **B** Cardiac magnetic resonance images of short-axis cine (**a**, **d**), T2-weighted (**b**, **e**), and late gadolinium enhancement (LGE) (**c**, **f**) images at the time of HCM diagnosis (**a**–**c**) and after resuscitation (**d**–**f**). **a**, **d** Left ventricular wall thickness exhibited heterogeneity, with a maximum thickness of 15 mm. No change in the degree of wall thickening was observed. **b**, **c** There was no myocardial edema. (e and f) Areas with high T2 signals were observed in the inferoseptal area and anterolateral wall (**e**) and corresponded to those of LGE (arrow).

After resuscitation, whole-exome sequencing was performed after obtaining written consent from the patient and her parents. A heterozygous missense variant, c.583A>T (p.Ile195Phe), was detected in exon 8 of *TNNI3* (NM_000363.5), which encodes troponin I, a cardiac sarcomere component and subtype of troponin. This variant was not previously reported in the Genome Aggregation Database (gnomAD: URL: http://gnomad.broadinstitute.org/) or in the Japanese genome databases jMorp^3^ and HGVD^8^. This variant was absent in her healthy parents, indicating a de novo occurrence (Fig. 2A). In silico analysis was used to predict the effects of the variant on protein function using a combination of Alpha Missense^9^ and dbNSFP^10^. The Alpha Missense score was 0.9259, suggesting a likely pathogenic nature. The CADD score was 23.3, the SIFT score was 0.001 (damaging), and the FATHMM score was 0.891 (damaging), all indicating potential pathogenicity. Approximately 80% of reported pathological variants of *TNNI3* are located in exons 7 and 8, which encode the domains that interact with myocardial actin and cardiac troponin C, which are sarcomere components^11^. *TNNI3* c.583A>T (p.Ile195Phe) was also located in exon 8 (Fig. 2B)^12^. Thus, the variant identified in this study can be classified as a likely pathogenic variant because it met the PS2, PM1, PM2, and PP3 criteria of the American College of Medical Genetics and Genomics/Association for Molecular Pathology (ACMG/AMP) guidelines^13^.

**Fig. 2:**
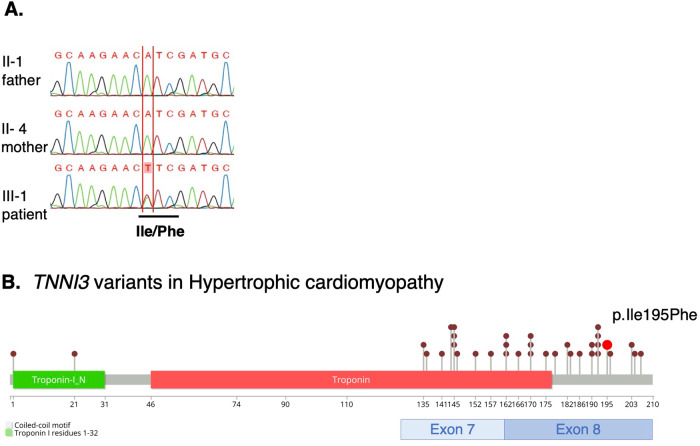
Genetic analysis of the family. **A** Sanger sequencing results for the TNNI3 (NM_000363.5) gene in the parents (II-1, II-4) and the patient (III-1). The patient carries a heterozygous missense variant, c.583A>T, p.Ile195Phe. **B** *TNNI3* pathogenic variants associated with HCM previously reported in ClinVar (URL: https://www.ncbi.nim.nih.gov/clinvar/) are shown in the form of a lollipop plot. The red plot represents the variant identified in this patient, located in exon 8.

The calcium sensitivity of myocardial contraction is regulated by the troponin complex, which comprises three subunits: troponin I, troponin C, and troponin T. *TNNI3*, which encodes cardiac troponin I, the inhibitory component of myocardial contractility, plays a key role in regulating myocardial contraction and relaxation in response to fluctuations in intracellular calcium levels^4^. The prevalence of pathological variants of *TNNI3* is reportedly less than 5% in patients with cardiomyopathies, with a relatively low penetrance of approximately 50%^5,6^. However, its phenotype presents challenges for prognosis and risk assessment owing to the heterogeneity of the onset times and phenotypes even within the same family. Patients with pathological variants of *TNNI3* reportedly experience severe clinical outcomes, such as fatal arrhythmias and sudden death, even in children^7,14^.

This case involved a patient with de novo pediatric HCM who experienced fatal arrhythmia during the follow-up period, necessitating the insertion of an ICD for secondary prevention after resuscitation. Accurately predicting disease severity solely based on clinical examination at the time of diagnosis remains challenging. Pediatric HCM, although less commonly diagnosed in clinical practice, is associated with a more severe disease status than adult HCM^15^.

HCM is one of the major causes of sudden cardiac death in childhood^7,11^, and individuals diagnosed with HCM during childhood have a poorer prognosis than those diagnosed in adulthood^16^. Moreover, 90% of pediatric HCM patients who undergo ICD insertion have been reported to have de novo variants^6^. De novo variants are a major cause of severe early-onset genetic diseases^17^. Genetic testing plays a crucial role in identifying clinically diagnostic genetic factors associated with HCM, particularly in young populations. However, genetic testing typically focuses only on proband analysis, and comprehensive family genetic evaluation is still underutilized in clinical practice, potentially leading to failure in identifying pathogenic variants. Thus, in de novo pediatric HCM patients, variants identified in disease-related genes through family genetic testing may have a significant influence on phenotypic development.

Although patients with HCM have multiple potential life-threatening factors that contribute to disease severity and sudden cardiac death, identifying high-risk patients, particularly children with HCM, remains challenging^16^. Therefore, genetic testing within families can be useful for risk stratification and treatment optimization, especially for preventing sudden cardiac death in pediatric HCM patients.

## HGV Database

The relevant data from this Data Report are hosted at the Human Genome Variation Database at 10.6084/m9.figshare.hgv.3382.
